# Alterations in the basal ganglia in patients with brain tumours may be due to excessive iron deposition

**DOI:** 10.3892/ol.2014.2638

**Published:** 2014-10-24

**Authors:** VÍT HERYNEK, DITA WAGNEROVÁ, ALBERTO MALUCELLI, JOSEF VYMAZAL, MARTIN SAMEŠ, MILAN HÁJEK

**Affiliations:** 1 Department of Diagnostic and Interventional Radiology, Institute for Clinical and Experimental Medicine, Vídeňská, Prague 14021, Czech Republic; 2 Department of Neurosurgery, JE Purkyně University and Masaryk Hospital, Sociální péče, Ústí nad Labem 40113, Czech Republic; 3 Department of Radiology, Na Homolce Hospital, Roentgenova, Prague 15030, Czech Republic

**Keywords:** magnetic resonance imaging, brain tumours, basal ganglia, iron, oxidative stress

## Abstract

The accumulation of iron in the brain is a common physiological process. However, alterations in the deposition of iron or other paramagnetic substances are associated with various diseases. In the present study, the deposition of paramagnetic substances in patients with brain tumours was evaluated using T_2_ relaxometry. A total of 23 patients with untreated tumours or with recurrent tumours following treatment, together with a group of 19 age-matched healthy controls, were examined using T_2_ relaxometry at 3T. The relaxation times in the basal ganglia, thalamus and white matter were evaluated. Significantly lower T_2_ relaxation times were identified in the basal ganglia and thalamus of the patients with tumours, as compared with those of the controls (P<0.05). No statistically significant difference was identified between patients with untreated or recurrent brain tumours. The reduction in T_2_ relaxation times in the brain tumour patients was possibly caused by the accumulation of iron, since iron homeostasis is known to be altered in patients with tumours. We propose that increased iron deposition is a consequence of a higher risk of oxidative stress caused by an increased iron concentration in the plasma or cerebrospinal fluid.

## Introduction

The accumulation of non-haemic iron in the brain, particularly in the basal ganglia (BG), is a common process initiated shortly following birth (1). During the first two decades of life, the iron concentration in the BG increases rapidly and then the accumulation slows down, and the concentration of iron in the BG follows an approximately exponential association curve. Alterations in the deposition of iron and other paramagnetic substances have been associated with various diseases. Increased deposition of paramagnetic substances in the brain has been observed in patients with liver diseases (2), psychiatric (3) and neurodegenerative disorders (4,5).

Although iron is an essential element in human nutrition, free iron (Fe^2+^) is closely associated with the production of reactive oxygen species (ROS), which may induce biological damage through oxidative stress. In the Fenton reaction, Fe^2+^ reduces H_2_O_2_ and subsequently produces a hydroxyl radical (•OH). Increased iron deposition in ferritin molecules is therefore considered to be a protective mechanism. The Fe^2+^ ions are trapped by ferritin, oxidised to Fe^3+^ and safely stored inside the protein molecule.

However, increased iron stores may be associated with an elevated risk of cancer. The association between iron and carcinogenesis has been recognised for a number of years (6). Increased body iron stores (indicated by higher serum ferritin levels and elevated transferrin concentrations) have been associated with malignant neoplasms (7) and leukaemia (8). Animal experiments as early as 1959 revealed that repeated injections of iron induce malignant tumours (9). Iron may be involved in the initiation or promotion of colorectal, liver, kidney, lung, stomach and other types of cancer (10).

In addition, increased concentrations of an iron-storage protein, ferritin, have been detected in the cerebrospinal fluid (CSF) of patients with glioblastoma (11). The authors ascertained the presence of ferritin in tumour cells in one patient and hypothesised that CSF ferritin was secreted by the tumour cells. The transfer of ferritin from the serum through the blood-CSF barrier does not occur on a large scale, due to the high molecular weight of ferritin and its hydrodynamic radius.

Attempts have been made to reduce the impact of iron redox activity during cancer treatment through the chelating of surplus iron ions (12); however, the alterations in Fe metabolism in cancer are not yet fully understood. Changes may occur during iron absorption, iron transport and iron storage (7,8,11,12). Tumour cells are also known for the upregulation of transferrin receptor expression on the cell surface (12).

Magnetic resonance imaging (MRI) is sensitive to the presence of paramagnetic ions in tissues. Alterations in paramagnetic or superparamagnetic ion concentrations manifest as a hyperintense signal on T_1_-weighted images or as a hypointense signal on T_2_-weighted images, as the ions substantially shorten relaxation times. Iron in iron-storage molecules substantially shortens T_2_ due to its magnetic properties. The most common type of iron-storage molecule, ferritin, which is soluble, also exerts a clear effect on the T_1_ relaxation time, whereas insoluble pathological hemosiderin markedly influences T_2_, but has no significant effect on T_1_ (13). The changes in relaxation times may be quantified by MR relaxometry (14).

In the present study, the deposition of paramagnetic substances in patients with brain tumours was retrospectively evaluated using T_2_ relaxometry.

## Materials and methods

### Patients

A total of 23 patients (mean age 46±12 years) were examined at the Department of Diagnostic and Interventional Radiology, Institute for Clinical and Experimental Medicine (Vídeňská, Czech Republic). The patients were divided into two groups. Group 1 consisted of 12 subjects (mean age, 46±13 years) with an untreated tumour in the brain: Six subjects with high-grade gliomas (HGG), four subjects with low-grade gliomas (LGG) and two subjects with lymphomas (Lym). Group 2 consisted of 11 subjects (mean age, 46±11 years) with tumour recurrence following treatment (chemotherapy and radiotherapy and/or resection). The primary tumour was HGG in six subjects and LGG in five subjects. All patients involved in the study had the diagnosis verified by histological methods. The control group consisted of 19 age-matched healthy patients (mean age, 47±12 years). All patients were informed with regard to the examination procedure and provided an informed consent approved by the local ethical committee. Clinical procedures were certified according to the ISO 9001:2008 norm.

### MRI

**Figure 1:**
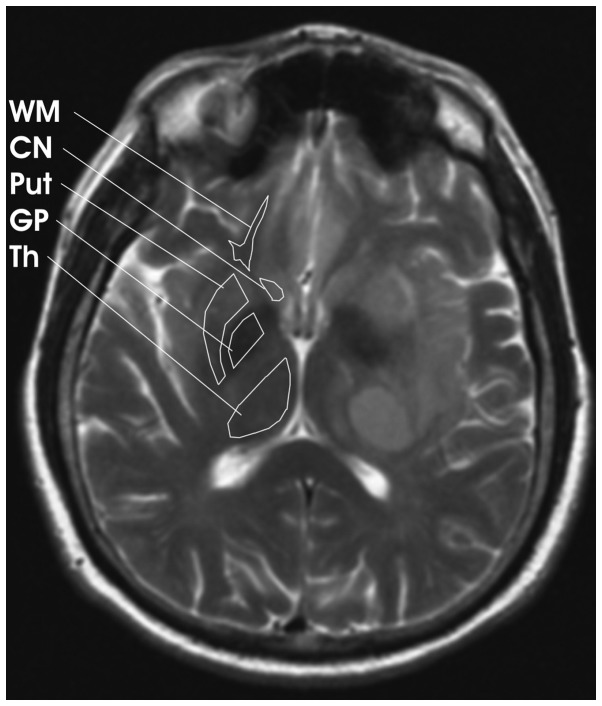
A T_2_-weighted magnetic resonance image of a patient with a high-grade glioma IV (glioblastoma multiforme in the left hemisphere). The regions of interest in the white matter (WM), caudate nucleus (CN), putamen (Put), globus pallidus (GP) and thalamus (Th) contralateral to the lesion are highlighted.

A 3 Tesla clinical MR imager Magnetom Trio (Siemens, Erlangen, Germany) equipped with a transmit/receive (Tx/Rx) head coil was used. A standard imaging procedure consisting of native T_1_- and T_2_-weighted images and contrast-enhanced T_1_-weighted images (in the patient group only) was supplemented with a Carr Purcell Meiboom Gill sequence (32 echoes, echo-spacing echo time = 13.2 ms, repetition time = 3,000 ms and slice thickness = 5 mm) for T_2_-mapping. A slice containing the BG was evaluated. T_2_ maps were calculated using custom-made ViDi software (Department of Diagnostic and Interventional Radiology, Institute for Clinical and Experimental Medicine) utilising a three-parameter fit (15). T_2_ values were then obtained from the globus pallidus (GP), putamen (Put), caudate nucleus (CN), thalamus (Th) and frontal white matter (WM) in the two hemispheres. However, tumours are commonly accompanied by extensive oedema, which may significantly prolong the T_2_ relaxation time in BG. This influence in the ipsilateral hemisphere cannot be separated from other changes that affect T_2_ relaxation, thus, the T_2_ values were substantially dispersed. Therefore, for further evaluation and comparison, only values found in the contralateral hemisphere were used (Fig. 1). Infiltration of the tumours into the contralateral hemisphere was not observed in the selected patients.

### Statistical analysis

Student’s t-test was used for comparing the relaxation times of the different patient groups and the controls. P<0.05 was considered to indicate a statistically significant difference. The equality of variances was analysed using an F-test. For unequal variances, a variant of the t-test for unequal variances was employed; this was the case for data obtained from the WM.

## Results

### T_2_ relaxation times in patients with untreated and recurrent, treated brain tumours

Significantly lower T_2_ relaxation times were identified in the GP, Put, CN and Th of brain tumour patients as compared with those of the controls (P<0.05, Table I). In the group of patients with untreated brain tumours, the T_2_ in the GP, CN and Th was significantly reduced, as compared with the controls (P<0.05). However, the difference in the Put T_2_ values was not statistically significant due to the high data dispersion. In the patients with recurrent tumours who had received treatment, significantly lower T_2_ values in the GP, Put, CN, Th and WM were detected, as compared with the controls (P<0.05).

No statistically significant differences were identified between patients with untreated tumours and those with recurrent tumours in any of the examined structures. However, a trend toward lower T_2_ values in the patients with recurrent tumours was detected, which was verified by a paired t-test between the average values in the given structures in the two patient groups.

No difference in the WM T_2_ values between the group of patients with untreated tumours and the control group was observed; however, a significant difference in these values between the group of patients with recurrent tumours and the control group was identified (P<0.05).

### T_2_ relaxation times in patients with different types of brain tumour

**Figure 2:**
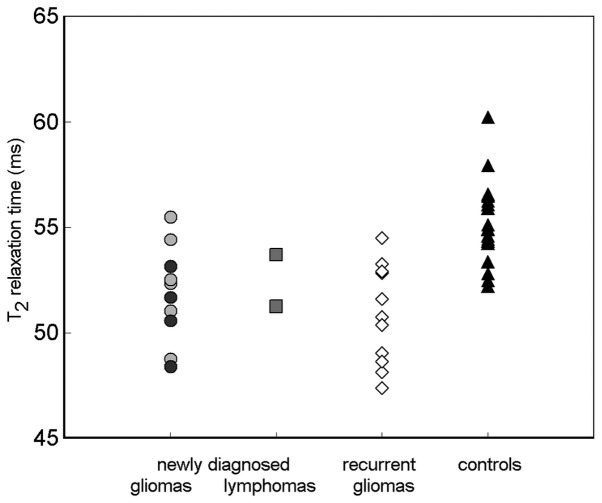
Distribution of T_2_ values in the contralateral globus pallidus in patients with newly diagnosed gliomas (high-grade glioma, light grey ●; low-grade glioma, dark grey ●), lymphomas (□) and recurrent gliomas (⋄), and controls (▲).

The graph in Fig. 2 shows the distribution of T_2_ values of GP in all investigated groups. The two patients with Lym are presented separately. No statistically significant differences were identified between subjects with different grades of newly diagnosed gliomas (HGG and LGG) or those with Lym. However, the numbers of subjects in these subgroups were too low for meaningful statistical analysis.

## Discussion

MRI is an *in vivo* imaging method sensitive to the paramagnetic ion content in tissues. In addition to T_2_ mapping, other potentially more sensitive methods, such as T_2_^*^ mapping or susceptibility weighted imaging (SWI), may be of interest and may be examined in a prospective study. However, the length of the procedures in the present study did not permit the addition of these sequences. Although the T_2_ relaxation time reflects the tissue structure and the water content, in addition to the presence of paramagnetic ion, which renders interpretation more difficult, this measurement is less sensitive to possible artefacts resulting from external field heterogeneities than T_2_^*^ mapping or SWI, and is a robust and effective technique. In addition, T_2_ mapping was included in our standard imaging procedure and the majority of the subjects were evaluated retrospectively, therefore alterations to the method were not possible.

The reduction in the T_2_ relaxation time in the BG observed in the present study may be caused by paramagnetic ion accumulation. A similar effect (i.e. T_2_ shortening) has been observed in a range of psychiatric and neurodegenerative diseases, and has been predominantly associated with iron accumulation (3,16). The trend detected in the present study towards lower T_2_ values in the patients with recurrent tumours, as compared with the patients with newly diagnosed lesions, may indicate the worsening of the patient’s state with time and disease progression, or the possible further damage to the tissue caused by radio/chemotherapy.

Although relaxometry is completely unspecific, the element responsible for T_2_ shortening is hypothesised to be iron, which is closely associated with cancer initiation (6,10) and tumour growth (17). Iron, an essential element for human life, may also be a carcinogenic agent (6), which has already been demonstrated in hepatocellular carcinoma (18) and breast cancer (19). Divalent iron contributes to the formation of ROS, which may cause damage to the DNA resulting in mutations and the initiation of cancer growth. Iron is also required for cellular proliferation due to its role in the active sites of a wide range of proteins involved in energy metabolism, respiration and DNA synthesis.

The reduction in T_2_ (hypothetically caused by an increased iron concentration) in the BG and Th observed in the present study may be an indirect consequence of tumour development rather than direct damage. Direct damage to the BG tissue by the active tumour is improbable; reduced T_2_ values contralateral to the tumour were observed and no changes were detected in the WM in the case of untreated tumours, although the majority of the tumours had affected the WM. Lower T_2_ values in the WM of patients with recurrent tumours (not observed in the patients with newly diagnosed tumours) may be a consequence of the damage to the tissue due to the therapy undergone. The observed changes in the BG are possibly the result of a general systemic effect of the tumours.

Although the concentration of the iron-storage protein ferritin in CSF in patients with glioblastoma has been found to be high, as compared with controls (11), the contribution of ferritin to T_2_ shortening may be disregarded. The reported mean concentration of ferritin in the CSF was 103 ng/ml (10.3 μg/100 g) (11), which is negligible, as compared with the concentration of non-haem iron in the BG, as reported in another study (21.3 mg/100 g fresh weight in the GP), the Th (4.76 mg/100 g fresh weight) or even the frontal WM (4.24 mg/100 g) (1).

The key issue is the transport of the putative iron into the BG or Th. In the present study, no signal enhancement to the contrast-enhanced MR images was observed in the BG; therefore, the blood brain barrier was presumed to be intact. Thus, the direct transport of ferritin molecules between the plasma and the tissue is improbable. However, previously identified elevated ferritin concentrations in the CSF indicate that the CSF may be an alternative route for iron transportation to the brain tissue (11).

As the tumour may be responsible for increased iron concentrations in the plasma (17), the transport may also be corrupted at the transferrin/transferrin receptor level. An increase in the concentration of ferrous ions inside the cell indicates the threat of oxidation stress induced by Fe^2+^. To protect the tissue, the ferrous ions are trapped and oxidised to ferric ions within the ferritin molecules and stored. This accelerated protective process may result in a substantially higher deposition of iron compounds in the BG or Th. This hypothesis is in accordance with the known role of iron in carcinogenesis and the finding that the maintenance of iron homeostasis by chelation slows tumour growth (19).

Notably, in the present study, similarly reduced T_2_ values were detected in the two patients with Lym and the patients with gliomas (Fig. 2), although the origin of Lym tumours is in the lymphatic tissue, in contrast to gliomas, which are intrinsic to the brain tissue. This finding corresponds to the hypothesis that increased iron accumulation is a reaction to localised alterations in iron homeostasis associated with tumour growth and angiogenesis rather than a consequence of direct changes in the brain tissue.

The present study demonstrated that the reduction in the T_2_ relaxation time in patients with brain tumours was possibly caused by the deposition of paramagnetic ions in the BG and Th. Non-specific changes in the T_2_ relaxation times in the BG occurred in patients with untreated tumours and those with recurrent tumours, regardless of tumour localisation. We hypothesise that the changes in the BG and Th are caused by iron deposition in ferritin molecules to eliminate excessive ferrous ions from the tissue in order to provide protection from oxidative stress.

Although the present study has no direct impact on current diagnosis or tumour treatment practices, the study contributes to the understanding of how brain tumours influence, through homeostatic changes, even distant healthy tissues.

## Figures and Tables

**Table I:** T_2_ relaxation times in the globus pallidus, putamen, caudate nucleus, thalamus and white matter of patients (contralateral to the lesion) and controls.

	T_2_ (ms)				
	
Patient group	Globus pallidus	Putamen	Caudate nucleus	Thalamus	White matter
All patients	51.4±2.2^a^	63.2±4.1^a^	73.4±4.8^a^	70.8±2.7^a^	69.2±4.6
Patients with an untreated tumour	52.0±2.0^a^	63.5±3.8	74.0±5.0^a^	70.6±2.4^a^	70.3±5.3
Patients with a recurrent tumour	50.9±2.3^a^	62.8±4.3^a^	72.9±4.5^a^	71.0±3.0^a^	68.0±3.3^a^
Controls	55.2±2.0	67.3±4.7	78.4±3.4	75.1±2.2	70.5±2.5

a P<0.05, as compared with the control group.
